# An appetite for research: an interview with Sadaf Farooqi

**DOI:** 10.1242/dmm.052379

**Published:** 2025-04-28

**Authors:** Sadaf Farooqi

**Affiliations:** ^1^ University of Cambridge, Institute of Metabolic Science, Addenbrooke's Hospital, Cambridge CB2 0QQ, UK; ^2^ NIHR Cambridge Biomedical Research Centre, Cambridge CB2 0QQ, UK

## Abstract

Professor Sadaf Farooqi is a clinician and researcher investigating the genetics underpinning obesity. Her research uncovered the first known genes that cause severe obesity, highlighting the significant role of appetite in regulating weight gain. Sadaf's work has been instrumental in proving that many observations in mice are also true in humans, paving the way for novel treatments and shifts in policy. After studying medicine at the University of Birmingham, Birmingham, UK, Sadaf completed her PhD on the genetics of severe childhood obesity at the University of Cambridge, Cambridge, UK, marking the beginning of her impressive research career in this field. She is currently Professor of Metabolism and Medicine at the University of Cambridge and Honorary Consultant in Diabetes and Endocrinology at Addenbrooke's Hospital, Cambridge, UK. Sadaf previously served on the Board of Directors for Disease Models & Mechanisms' publisher, The Company of Biologists. Here, we discuss the fascinating insights from her work on obesity and appetite, her approach to exploring new research questions, and how these discoveries can ultimately impact patients and society.


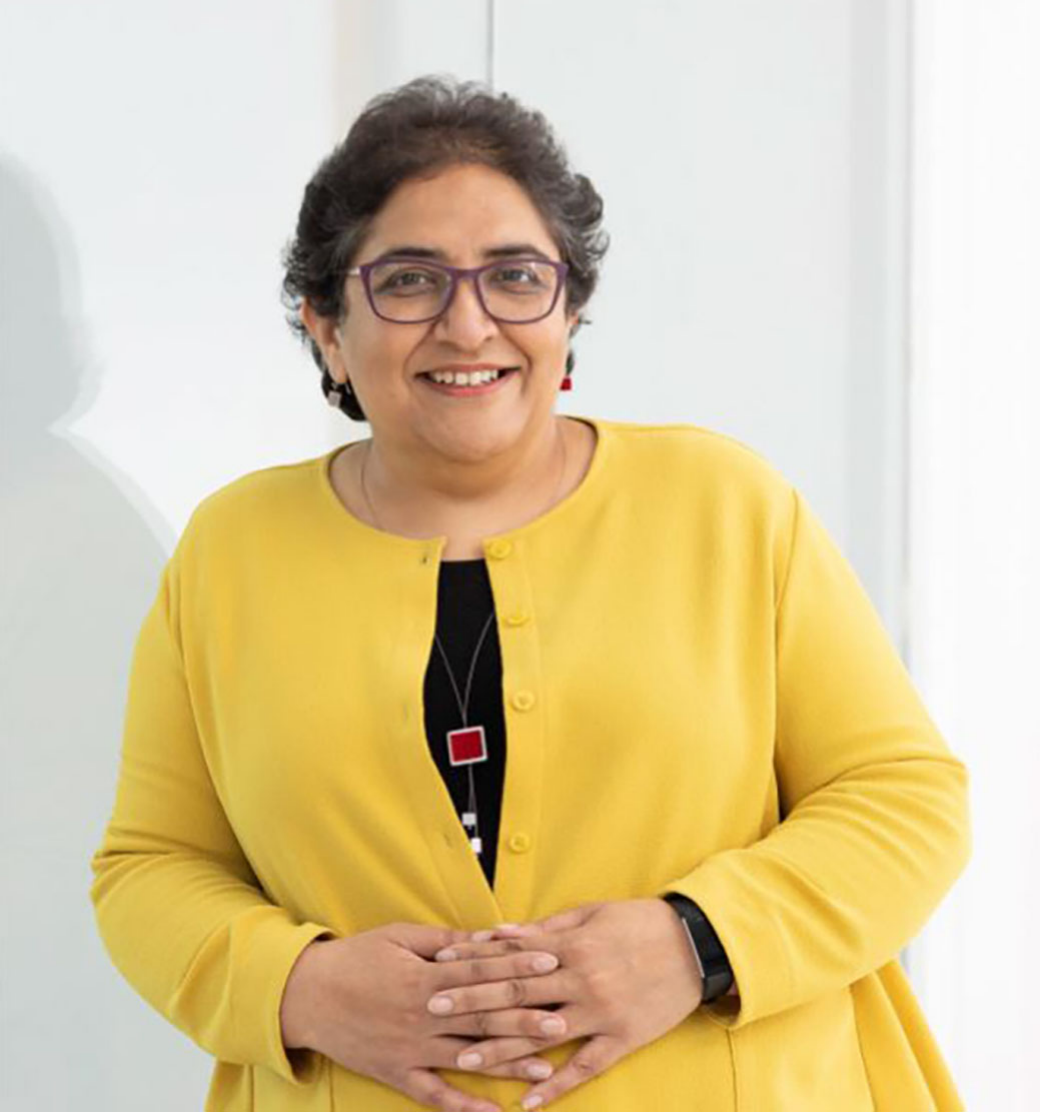


**Sadaf Farooqi**

**What made you want to become a researcher after you studied medicine?**

I was fortunate to have exposure to research very early on during my medical training. I did some research into cot death, which was one of the major causes of mortality in infants at the time. This was back in the late 1980s in the UK, and I actually made a pretty key observation as a student, which ended up changing policy and was published in The Lancet (Farooqi et al., 1991). This got me very excited about research and the way that it could make a difference to many people's lives.

**How does your perspective as a clinician shape your research?**

I always think it's a great privilege to be a doctor, and I think it's an even greater privilege to be both a doctor and a scientist. My research is very much shaped by my patients. Then, once you get into the research, you find fascinating new things that you never thought you'd be thinking about, so the pursuit of science and identifying interesting questions to tackle becomes the focus itself. But the direction of travel for me is always about finding ways to benefit patients.

**You have done extensive studies in patients to identify rare genetic variants associated with severe obesity. What are some of the most interesting findings from this work?**

Initially, just being able to show that a variant in a single gene is sufficient to cause obesity in a person was the key early finding we made back in 1997 (Montague et al., 1997). That was also key to proving that the hormone leptin is a critical regulator of weight. Without any leptin, children will invariably develop severe obesity through failure to control their appetite: lack of leptin causes this intense drive to eat, or hyperphagia. This taught us that our appetite and our eating behaviour has a strong biological basis, something that hadn't really been proven in humans before that. That finding then paved the way for many subsequent studies that looked at several different genes expressed in the hypothalamus in the brain affecting pathways that regulate weight. We showed that disruption of those genes can cause obesity. Collectively, that work has given us an understanding of how body weight is regulated in the hypothalamus. It's shown us that genes can contribute to obesity and to weight regulation, and this has informed diagnostic testing in people around the world. It's also provided the evidence base for developing drugs that can target these pathways. Now, we're very excited that we can actually give drugs to people with some of these genetic conditions and often entirely treat their severe obesity.

One of the things that we found was that the leptin–melanocortin pathway is key to regulating different aspects of eating behaviour. It affects how much pleasure you get from food. In animals, there was very good evidence that it affects food preference, e.g. the type of food the animal will go for – fat versus carbohydrate. We thought, “wouldn't it be fascinating to test whether the same happens in human beings?”. There had been a few small studies with flavoured liquids, but not with meals, so we designed an experiment in humans in which we varied the fat content in a meal (van der Klaauw et al., 2016). This is quite hard, because if you have something very high in fat, it often becomes very oily, so you must make sure that it doesn't look different from the other meals. It doesn't work for spaghetti Bolognese, for example, because all the oil floats to the top. By a lot of experimentation in the kitchen, we figured out that chicken korma and rice would work well, and we could provide three meals with completely different fat content (20, 40 or 60%) that were identical in appearance and taste so nobody could tell them apart. We showed that people with the melanocortin 4 receptor (*MC4R*) variant ate over 90% of their food from the high-fat option. Even though the total amount people ate was very similar, people with the *MC4R* variant preferred the high-fat meal. We weren't sure if it was because fat is more palatable, so we did a second study varying sucrose concentrations across otherwise identical Eton mess desserts (meringue, cream and berries). Most people preferred a sweeter dessert, but not the people with the *MC4R* variant. Somewhat paradoxically, they didn't want the sweet food. Around the same time, people had done studies in mice that showed exactly the same thing. Remarkably, in mice, genetic or pharmacological inhibition of the same pathway increases preference for fat and decreases preference for sugar, so it's highly conserved between mice and humans. This pathway is there to defend against starvation: if you're starving, it makes more sense to eat fat, because you'll get twice as many calories from fat as you do from carbohydrate, and you can store the fat easily. We think that's why this pathway has evolved to affect multiple different components of eating behaviour.

[…] our work shows just how relevant and important mice and other model organisms can be to the study of human physiology and disease.

**This conservation between mouse and human eating behaviours is fascinating, and your work has proven that many observations in mice are also true for humans. How important have mouse studies been to uncovering pathways regulating body weight in humans?**

As people were beginning to unravel these pathways in mice, we were also able to explore their relevance in humans, and so the discoveries were really accelerated by working in mouse and human together. If anything, our work shows just how relevant and important mice and other model organisms can be to the study of human physiology and disease. We need to use model organisms because obesity is a disorder of systems, not of a single cell type. Now that we're at a more advanced stage in the field, mice are still hugely relevant, particularly for studying the brain. We can do some things in humans with imaging, for example, but we can't directly study neural circuits and understand synapses and how they function.

The mouse seems particularly good for obesity research because of these conserved pathways. It's less good for other things like lipid metabolism and even glucose homeostasis, but it's very good for studying the regulation of appetite and eating behaviour. Fascinatingly, many of these pathways also affect other behaviours, like socialisation, anxiety and aggression, which are also highly conserved across species because they're fundamental instinctive behaviours.

**You have been involved in several clinical trials for obesity treatments. What aspects are most important to consider when trying to help new treatments reach patients?**

Some of the work that we have done has been picked up by colleagues or companies to make drugs. We've built up a body of knowledge on these drugs and targets, but we also have the advantage of having a lot of patients in a very organised cohort. We've been working with them for many years, so we're ideally placed to translate the findings and run the trials. The decision about when you might undertake a trial is normally based on having sufficient evidence in animal models about how the drug might work, whether it's safe, whether toxicity has been considered – and then you have to make sure it's suitable to give to people. After phase 1 studies have shown that the drug is safe, you do the phase 2 study to test it in people with a particular condition. Of course, I would like to have things as soon as possible, because I know my patients are in a lot of need. Understandably, we have to make sure that what we give them is safe and has been properly developed and tested. It's about engaging with the people doing the science at an early stage and working with them to try to accelerate things. I'm now working with quite a lot of companies who are developing new medications and engaging with people at an earlier stage so that the whole plan for the drug development is informed by the needs of the patients from the beginning.

**How is the increased availability of new weight loss drugs like Ozempic and Wegovy impacting the field?**

It's really exploded the field in many ways. As a doctor, for about 25 years, I've had either no drugs or maybe one drug that I could give people with severe obesity. The fact that there are actually drugs that can work is amazing. It's a little bit challenging, because of the way that it's exploded and that everybody knows about these drugs. They have been somewhat hijacked through use by media personalities and others who don't necessarily need the treatment – this has had a complex effect on how they're viewed. But if you think purely about people with severe obesity who have major health problems, they clearly have a need for medication to help them lose weight. It's great that we now have some medications, and there are many more coming down the line, which I'm very pleased about. But we need to make sure they're available to the people who need them, as opposed to the people who would like to have them, say, to lose a few pounds before the Oscars ceremony. I think it's really important that we address the clinical need and don't get sidetracked by these personalities and the abuse of the drugs. Access to healthcare is a big issue. In the UK, for example, these drugs aren't being delivered to enough people through the National Health Service so some people are paying privately – but many people who really need the drug can't afford to pay privately. So that's a major issue that we're trying to tackle. But the drugs are effective, and they work on the brain circuits that are regulating appetite. We've contributed to understanding some of those circuits, as have many others, particularly in work in mice, so it's great to be able to see that this knowledge has now led to effective drugs.

**You conducted research about how obesity influences the efficacy of COVID-19 vaccines during the height of the pandemic. How did you adapt your research during this period and what were your findings?**

We weren't able to continue working in the lab in the first phase of the pandemic, as was the case for many, but it was becoming rapidly clear that people with obesity were at much higher risk of infection with severe acute respiratory syndrome coronavirus 2 [SARS-CoV-2; the virus that causes coronavirus disease (COVID-19)] and were much more likely to die once they had the infection. And this was happening around the world. It became clear that this was a major public health crisis, and it was a question related to obesity. So, it seemed to me that, as a researcher studying obesity, I should want to understand something as important as that.

We started by speaking to our immunologist colleagues to figure out how our knowledge about obesity could help address some of these questions. We wanted to understand the susceptibility of people with obesity to COVID-19 as well as whether the vaccines were equally as effective in these people. That was a good question, because we knew from previously published studies that a range of vaccines are not as effective in people with obesity. This had gone a bit below the radar: there were a few papers about rabies vaccines and hepatitis vaccines given to some groups where they're ineffective if the person has a higher body mass index (BMI) and no one knows why. We thought, “okay, if this COVID-19 vaccine is not as effective in people with obesity, and people with obesity get worse infections and are more likely to die, that's a really big deal, so we better address this question”.

One of the challenges was that the baseline was moving the whole time. We set up to study people with obesity, then the national vaccination campaign was launched about a month after we started, so we had to move rapidly in order to capture people before they were vaccinated so that we could study them during the follow-up period. It was very exciting – I'm glad we mobilised when we did, because it was a one-off opportunity and we were able to address a very fundamental question. It's informed policy globally, because we can clearly see that people with obesity do have impaired vaccine responses as B-cell-mediated immunity wanes more rapidly. We were able to do two separate studies: one measured people over time and documented how their antibody titres declined, and the second was an epidemiological study using the extensive health record data captured in Scotland. We were able to look at a large number of records and see who was coming in with infections, how bad their infections were and whether there was a mortality. We could then link that to BMI, as well as whether they'd been vaccinated and the time since vaccination. Putting the pieces of the puzzle back together, we could effectively see that the antibody titres observed in the experimental study overlapped with the fact that there were more frequent admissions with infections in people with obesity. This is probably because the titres were waning, so they were being seen earlier with a secondary infection.

Our research has been a very useful way of communicating to public audiences that there is scientific proof of a genetic component to obesity that people have no control over.

**How can a better understanding of the genetic causes of weight gain influence public policy and help destigmatise obesity?**

One of the challenges is that, for a long, long time, obesity has been a rather unfashionable scientific area. People thought it was as simple as, “if you eat too much and don't do enough exercise, you gain weight”. What our work and the work of others has clearly shown is that it's not as simple as that. There's a lot of variability in how much weight you gain or don't gain. This is down to genetic factors, many of which we have identified. Our research has been a very useful way of communicating to public audiences that there is scientific proof of a genetic component to obesity that people have no control over. If you can show that disrupting a single gene causes obesity in a child, that's a very powerful way of explaining the contribution of genetics and biology to weight regulation. We've tried to use that knowledge to educate healthcare professionals, policy makers, politicians, etc. It can be quite challenging, because people have very firmly held views, often not informed by any science. But part of the job for all scientists should be to use our science to educate people more broadly. I think it's particularly challenging in this area, but important.

**What do you think is an important research question to address that may change your field going forward?**

One of the things that I'm very interested in is some work that we're doing to study thin people. We started this before we knew about some of these new medications, but I thought that one of the ways to try to find new mechanisms that could be targeted for therapy might be if you could understand how some people can eat what they like and stay thin. We all anecdotally know people like that, and it's always fascinated me how that happens. A few years ago, we recruited a cohort of healthy thin people who don't have a medical condition, don't have an eating disorder and don't exercise too much, but they're still very thin. We're now doing the genetic studies to try to figure out what genetic variants they are carrying that are keeping them thin. It's shown some really interesting things, including potential mechanisms that might be able to be targeted by new therapies. In the cohort, some of them have a small appetite, and so some of the genes are probably causing this, but there are also people who can eat what they like without putting on weight. There's clearly something going on with how they're using their calories at a metabolic level that's keeping them thin, which is what we're particularly keen to find out.

I think we should grasp every opportunity to try to deliver on things that can improve people's lives and further our understanding of science.

**What advice do you have to pass onto early-career researchers?**

I think you've got to be passionate about what you do. It's hard work doing research, whether it's medicine or any other area of science, but if you are interested and passionate about it then of course you want to work hard to understand that question. I think it's also important to consider what makes a good research area or project proposal – it's got to be an important question that means something to someone. Sometimes a timely question is important – that doesn't necessarily mean you have to do something just because it's fashionable, but sometimes a technique can crack open a field and enable us to understand a disease in a way that we never could before. Tenacity is also really important, because any scientist will know the main challenges, the failed experiments and things that don't work. Focus is important, but at the same time, being able to stand back and look at the question from other people's perspectives can bring you new insights. Science is incredibly exciting and fun, and we're very privileged to be able to do it. I think we should grasp every opportunity to try to deliver on things that can improve people's lives and further our understanding of science.

**What do you enjoy doing outside of your work?**

I like doing straightforward things like socialising and travelling. Even though I spend a lot of my time thinking about food and eating behaviour, I spend quite a lot of my free time thinking about cooking different recipes from around the world and entertaining guests.
